# Comparative transcriptome analysis of lufenuron-resistant and susceptible strains of *Spodoptera frugiperda* (Lepidoptera: Noctuidae)

**DOI:** 10.1186/s12864-015-2183-z

**Published:** 2015-11-21

**Authors:** Antonio Rogério Bezerra do Nascimento, Pablo Fresia, Fernando Luis Cônsoli, Celso Omoto

**Affiliations:** Departamento de Entomologia e Acarologia, Escola Superior de Agricultura “Luiz de Queiroz”, Universidade de São Paulo, Piracicaba, São Paulo 13418-900 Brazil

## Abstract

**Background:**

The evolution of insecticide resistance in *Spodoptera frugiperda* (Lepidoptera: Noctuidae) has resulted in large economic losses and disturbances to the environment and agroecosystems. Resistance to lufenuron, a chitin biosynthesis inhibitor insecticide, was recently documented in Brazilian populations of *S. frugiperda*. Thus, we utilized large-scale cDNA sequencing (RNA-Seq analysis) to compare the pattern of gene expression between lufenuron-resistant (LUF-R) and susceptible (LUF-S) *S.* larvae in an attempt to identify the molecular basis behind the resistance mechanism(s) of *S. frugiperda* to this insecticide.

**Results:**

A transcriptome was assembled using approximately 19.6 million 100 bp-long single-end reads, which generated 18,506 transcripts with a N50 of 996 bp. A search against the NCBI non-redundant database generated 51.1 % (9,457) functionally annotated transcripts. A large portion of the alignments were homologous to insects, with the majority (45 %) being similar to sequences of *Bombyx mori* (Lepidoptera: Bombycidae). Moreover, 10 % of the alignments were similar to sequences of various species of *Spodoptera* (Lepidoptera: Noctuidae), with 3 % of them being similar to sequences of *S. frugiperda*. A comparative analysis of the gene expression between LUF-R and LUF-S *S. frugiperda* larvae identified 940 differentially expressed transcripts (*p* ≤ 0.05, *t*-test; fold change ≥ 4). Six of them were associated with cuticle metabolism. Of those, four were overexpressed in LUF-R larvae. The machinery involved with the detoxification process was represented by 35 differentially expressed transcripts; 24 of them belonging to P450 monooxygenases, four to glutathione-S-transferases, six to carboxylases and one to sulfotransferases. RNA-Seq analysis was validated for a number of selected candidate transcripts by using quantitative real time PCR (qPCR).

**Conclusions:**

The gene expression profile of LUF-R larvae of *S. frugiperda* differs from LUF-S larvae. In general, gene expression is much higher in resistant larvae when compared to the susceptible ones, particularly for those genes involved with pathways for xenobiotic detoxification, mainly represented by P450 monooxygenases transcripts. Our data indicate that enzymes involved with the detoxification process, and mostly the P450, are one of the resistance mechanisms employed by the LUF-R *S. frugiperda* larvae against lufenuron.

**Electronic supplementary material:**

The online version of this article (doi:10.1186/s12864-015-2183-z) contains supplementary material, which is available to authorized users.

## Background

The fall armyworm *Spodoptera frugiperda* (Lepidoptera: Noctuidae) occurs mainly in tropical and subtropical regions [1], causing large losses to cotton and corn crops in the Americas [2]. Recent changes in the cropping system in the Brazilian savanah (*Cerrado*) by the integration of crops such as cotton, corn, soybean and millet have led to an increase in the population densities of *S. frugiperda* [3]. Control of *S. frugiperda* has been based on the use of genetically modified plants that express *Bt* (*Bacillus thuringiensis*) toxins and on the use of synthetic insecticides. However, the strong selective pressure caused by these control methods has led to an increase of *S. frugiperda* resistance to *Bt* toxins and to insecticides from different chemical groups [3, 4].

Chitin biosynthesis inhibitors act by interfering with the synthesis or deposition of chitin on the exoskeleton and on other chitinized structures of insects [5]. Given the specificity of their mode of action, this group of insecticides has been shown to have great potential in integrated pest management (IPM) programs because of its low toxicity to humans and higher animals [6]. The high pressure resulting from the widespread adoption of chitin biosynthesis inhibitors such as lufenuron for the control of insect pests in the Brazilian *Cerrado* has modified the susceptibility of *S. frugiperda* populations [7]. The observed reduction in the susceptibility of natural populations of *S. frugiperda* to chitin-synthesis inhibitors may indicate the evolution of resistance to this insecticide. Thus, measures that enable the preservation of the useful life of this molecule are necessary.

Currently, the main resistance mechanisms of *S. frugiperda* to insecticides involve mutations that lead to the insensitivity of target sites and/or alterations in the activity of enzymes involved with the detoxification or sequestration of xenobiotics [8–10]. Despite these findings, little is known about the gene expression profile of strains resistant to chitin biosynthesis inhibitors, and there are no studies for resistant strains of *S. frugiperda* to such products.

Next-generation sequencers (NGS), such as Solexa/Illumina™ (Illumina^©^), 454 (Roche^©^) and SOLID™ (Applied Biosystems^©^) platforms, generate a large amount of data [11] that allows for an in depth investigation of transcriptomes at a low cost and in a short period of time.

We characterized the larval transcriptome of *S. frugiperda* and compared the larval gene expression patterns between LUF-R and LUF-S strains as a step towards understanding the molecular mechanism(s) involved in *S. frugiperda* resistance to lufenuron in order to support the further development of rational, sustainable tools for pest and resistance management strategies.

## Results

### *De novo* assembly of a reference transcriptome

Sequencing of cDNA libraries in the Illumina HiScan 1000® platform generated 68,027,513 reads of approximately 100 bp, corresponding to 6,802,571,300 nucleotides. After selection of reads through quality filters, 23.2 % of the reads obtained were discarded due to their low quality scores (see Additional file 1).

Changes in the *k-*mer parameter led to changes in almost all observed variables (see Additional file 2). Thus, assemblies with a *k-*mer of 23, 25, 47, 53 or 55 were selected for performing the reference *de novo* assembly. Assemblies used 14,337,437 nucleotides, equivalent to 71.8 % of the bases submitted for analysis. These individual assemblies were merged in a single *de novo* reference assembly, which resulted in the generation of 18,506 transcripts with sizes ranging from 100 to 6,517 bp (see Additional file 3), with a mean length of 774.75 bp (see Additional file 4), a N50 of 996 bp and a N90 of 411 bp.

### Functional annotation

From the 18,506 transcripts obtained, only 51.1 % (9,457) were functionally annotated after a heuristic search against the NCBI non-redundant protein database (see Additional file 5). The similarity analysis yielded *e-*values from 10^−3^ to 10^−32^ for nearly 30 % of the mapped transcripts, while 24 % transcripts had even lower *e*-values, ranging from 10^−32^ to 10^−61^. The highest *e*-value significance scores were identified in 6 % of the transcripts, and ranged from 10^−148^ to 10^−177^ (see Additional file 5). Almost all of the alignments obtained were related to insects (see Additional file 6), and their majority (45 %) aligned against the silk moth *Bombyx mori* (Lepidoptera: Bombycidae), followed by the monarch butterfly *Danaus plexippus* (Lepidoptera: Nymphalidae) (20 %). The representatives of the genus *Spodoptera* (Lepidoptera: Noctuidae) observed in the analysis were *S. littoralis, S. exigua, S. litura* and *S. frugiperda*, corresponding to 10 % of the obtained alignments; *S. frugiperda* accounted for 3 % of them. Nearly two-thirds (6,078) of the 9,457 annotated transcripts and gene ontologies were distributed in 13 functional groups in the category of proteins that function as *cellular components*, 11 groups in *molecular functions* and 23 groups in *biological functions* (see Additional file 7).

### Differential gene expression between LUF-S and LUF-R *S. frugiperda* larvae

**Fig. 1:**
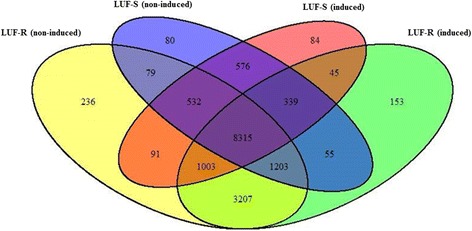
Distribution of differentially expressed transcripts from an RNA-seq analysis of susceptible (LUF-S) and lufenuron-resistant (LUF-R) strains of *S. frugiperda*, induced or non-induced by lufenuron

**Fig. 2:**
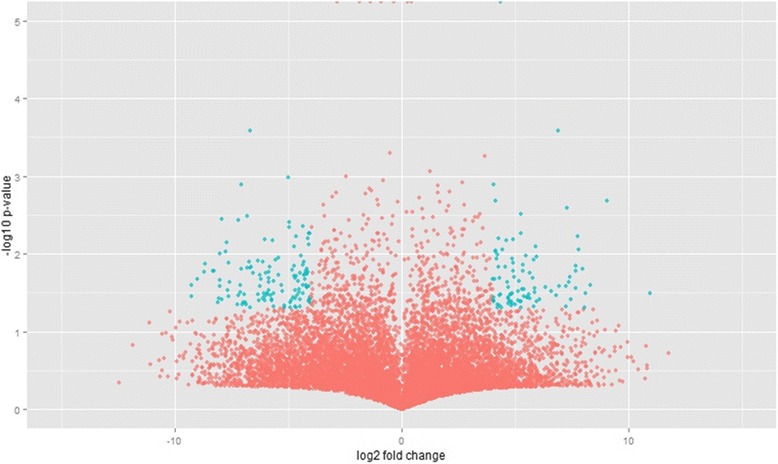
Distribution of the transcripts of the *S. frugiperda* transcriptome based on the comparative analysis of the gene expression of susceptible (LUF-S) and lufenuron-resistant (LUF-R) strains, induced or non-induced by lufenuron. Marked in green are the transcripts with a significant difference in the expression level, based on the discriminative significance values (*t*-test, *p* < 0.05) and relative expression (>4) adopted here

**Fig. 3:**
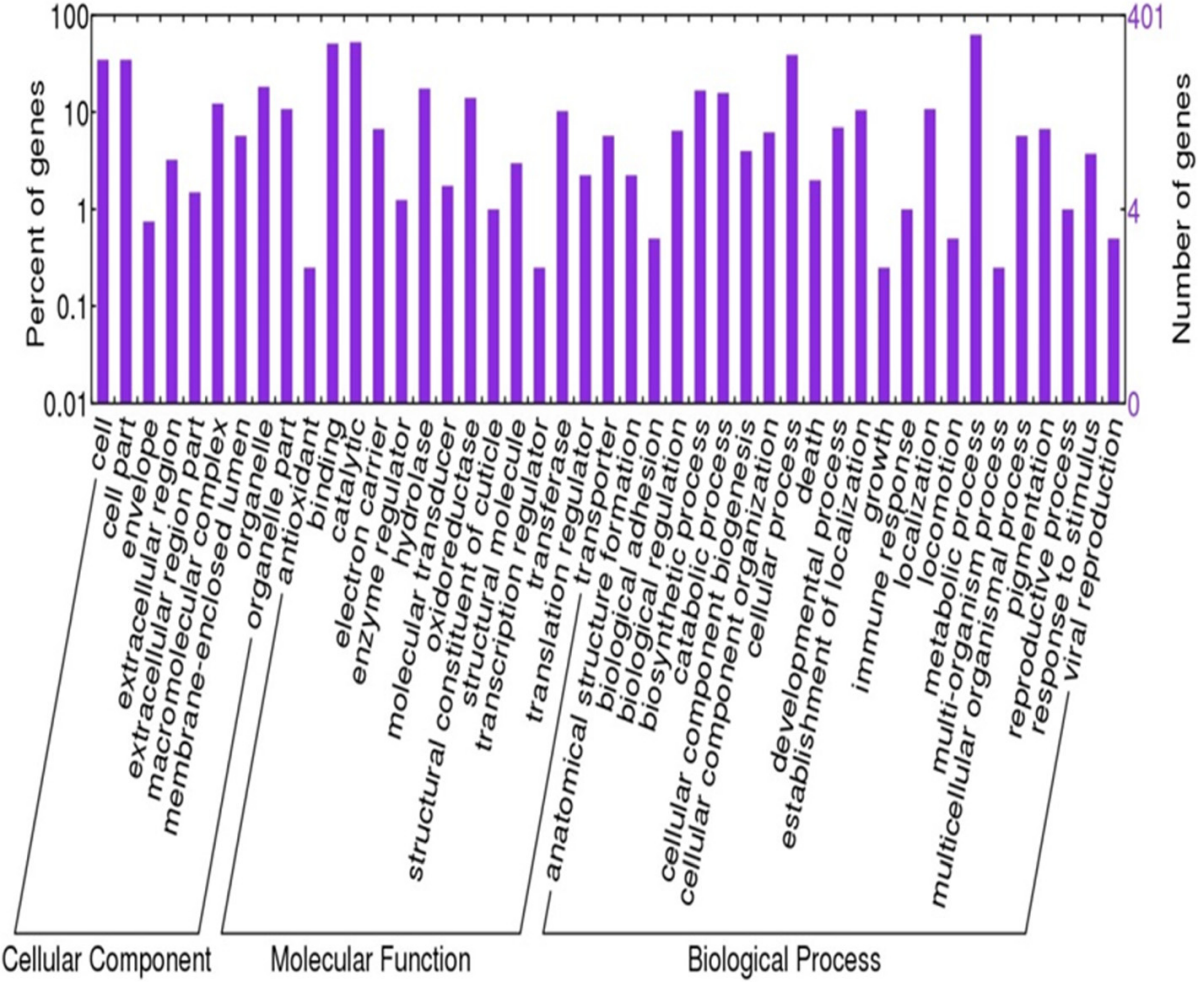
Distribution of gene ontology (GO) attributed to differentially expressed transcripts in susceptible (LUF-S) and lufenuron-resistant (LUF-R) *S. frugiperda* strains

A comparative gene expression analysis demonstrated LUF-R and LUF-S *S. frugiperda* larvae have distinct gene expression profiles (Fig. 1). Among the 18,506 transcripts evaluated, 940 were differentially expressed (*t*-test, *p* ≤ 0.05; fold change ≥ 4) (Fig. 2). These transcripts are from a large variety of functional categories, with a great number of transcripts associated with catalytic, binding and metabolic processes (Fig. 3). Among the differentially expressed transcripts, 25 transcripts were overexpressed in the larvae of the LUF-R strain, with fold change values greater than 100 times the expression of the larvae of the LUF-S strain. The fold change of the top 20 highly expressed transcripts in the LUF-R larvae ranged from 125 to 620 fold the expression in the LUF-S larvae. Conversely, eight transcripts were suppressed in the LUF-R strain, varying from −110 to −1,974 fold the expression in the LUF-S strain (see Additional file 8).

**Fig. 4:**
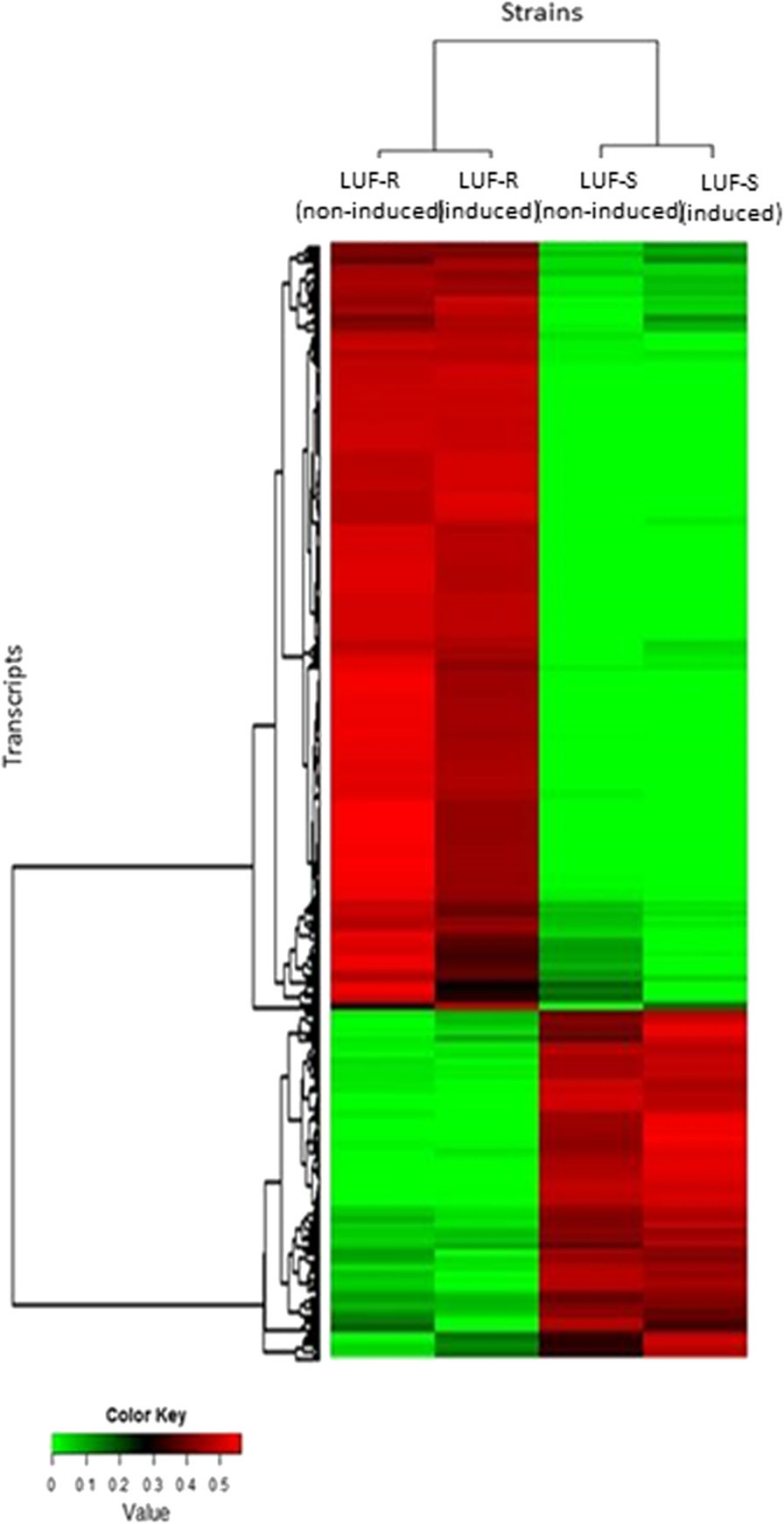
Comparative distribution of functionally annotated transcripts of lufenuron-susceptible (LUF-S) and lufenuron-resistant (LUF-R) *S. frugiperda* strains that showed changes in the expression level. RPKM values were represented as a scale of colors ranging from green to red, which will encompass values from the lowest (green) to the highest (red) RPKM values

The grouping analysis showed that the majority of the transcripts (61.3 %) were overexpressed, whereas 38.7 % were suppressed in the resistant strain when compared to the susceptible strain (Fig. 4). Our analysis did not indicate substantial differences in the pattern of expression within each strain (LUF-S and LUF-R) following their exposure to lufenuron, showing that the majority of the overexpressed and suppressed transcripts of the LUF-R strain exhibit constitutive expression.

**Fig. 5:**
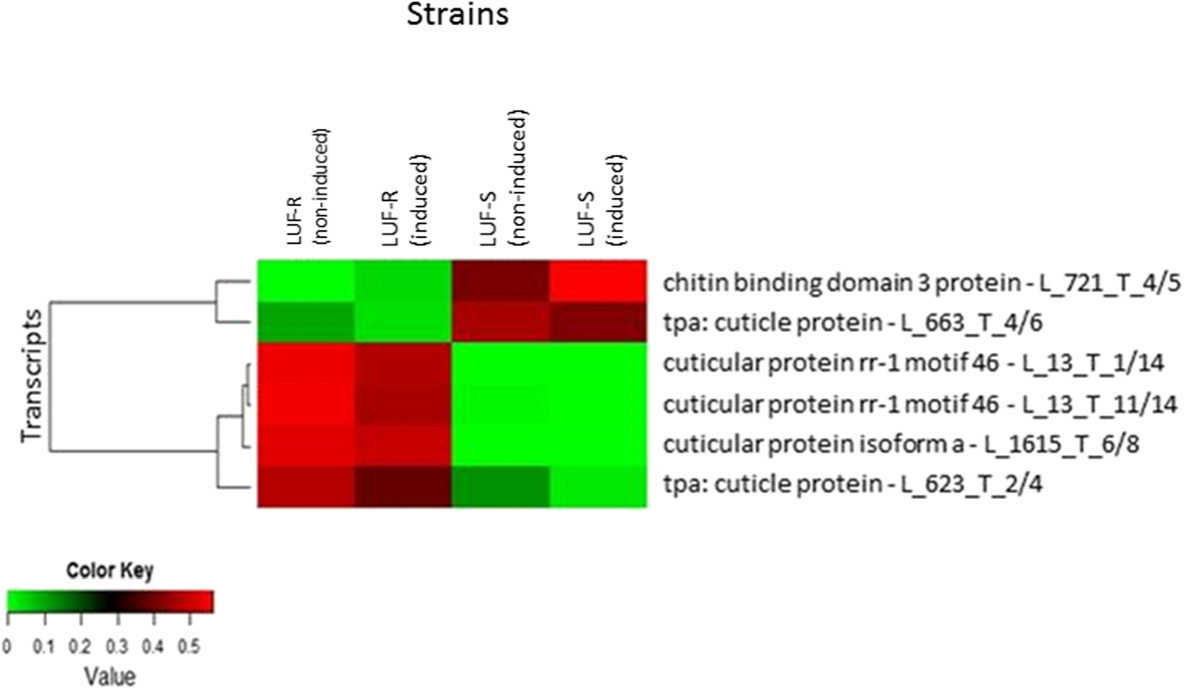
Comparative distribution of transcripts associated with cuticle metabolism of lufenuron-susceptible (LUF-S) and lufenuron-resistant (LUF-R) *S. frugiperda* strains that showed changes in the expression level. RPKM values were represented as a scale of colors ranging from green to red, which will encompass values from the lowest (green) to the highest (red) RPKM values

**Fig. 6:**
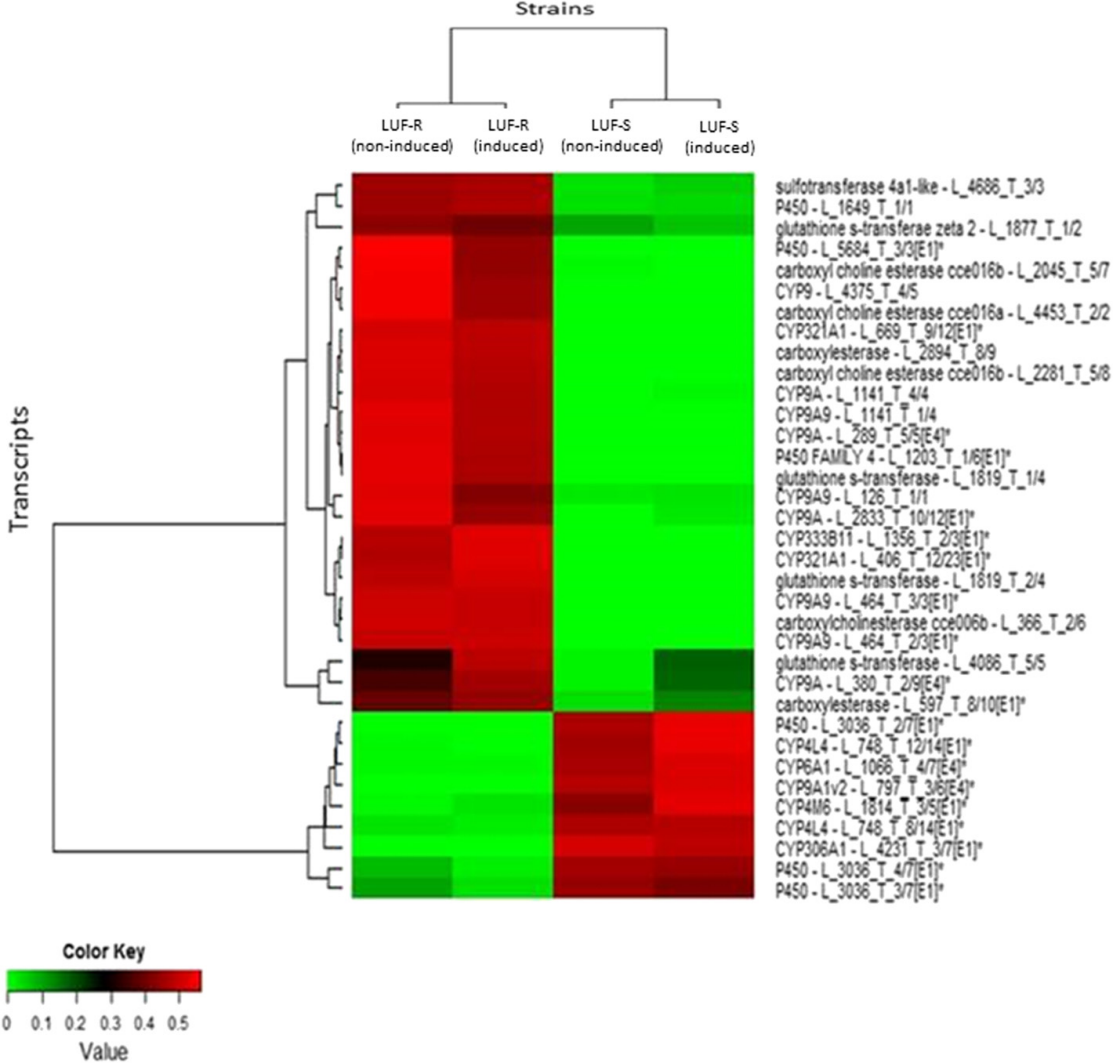
Comparative distribution of transcripts associated with detoxification enzymes of lufenuron-susceptible (LUF-S) and lufenuron-resistant (LUF-R) *S. frugiperda* strains that showed changes in the expression level. RPKM values were represented as a scale of colors ranging from green to red, which will encompass values from the lowest (green) to the highest (red) RPKM values. The title of each transcript consists of the identification of genes and code of the transcript. In P450’s transcribed capital letter and number is the class ID and group associated with gene superfamily

Among the differentially expressed genes, two groups of candidate transcripts were selected because of their association with the cuticle metabolism and detoxification processes, as they could be related to the resistance mechanism of *S. frugiperda* to lufenuron (Figs. 5 and 6). Two out of the six selected transcripts involved in cuticle metabolism (L_663_T_4/6 and L721_T_4/5) were suppressed, while the remaining was overexpressed in the LUF-R as compared to LUF-S larvae (Fig. 5).

Out of the 35 differentially expressed genes involved in detoxification processes, 24 of them were annotated as P450 (CYP) monooxygenase, four as glutathione-S-transferase (GST), six as carboxylase (CCE) and one as sulfotransferase (SUR). CYP was represented by several family members, especially CYP9A, CYP3, CYP4 and CYP6. Transcript levels of 26 of these genes were much higher in LUF-R larvae, while nine of them had their expression drastically reduced (Fig. 6).

### qPCR analysis

**Fig. 7:**
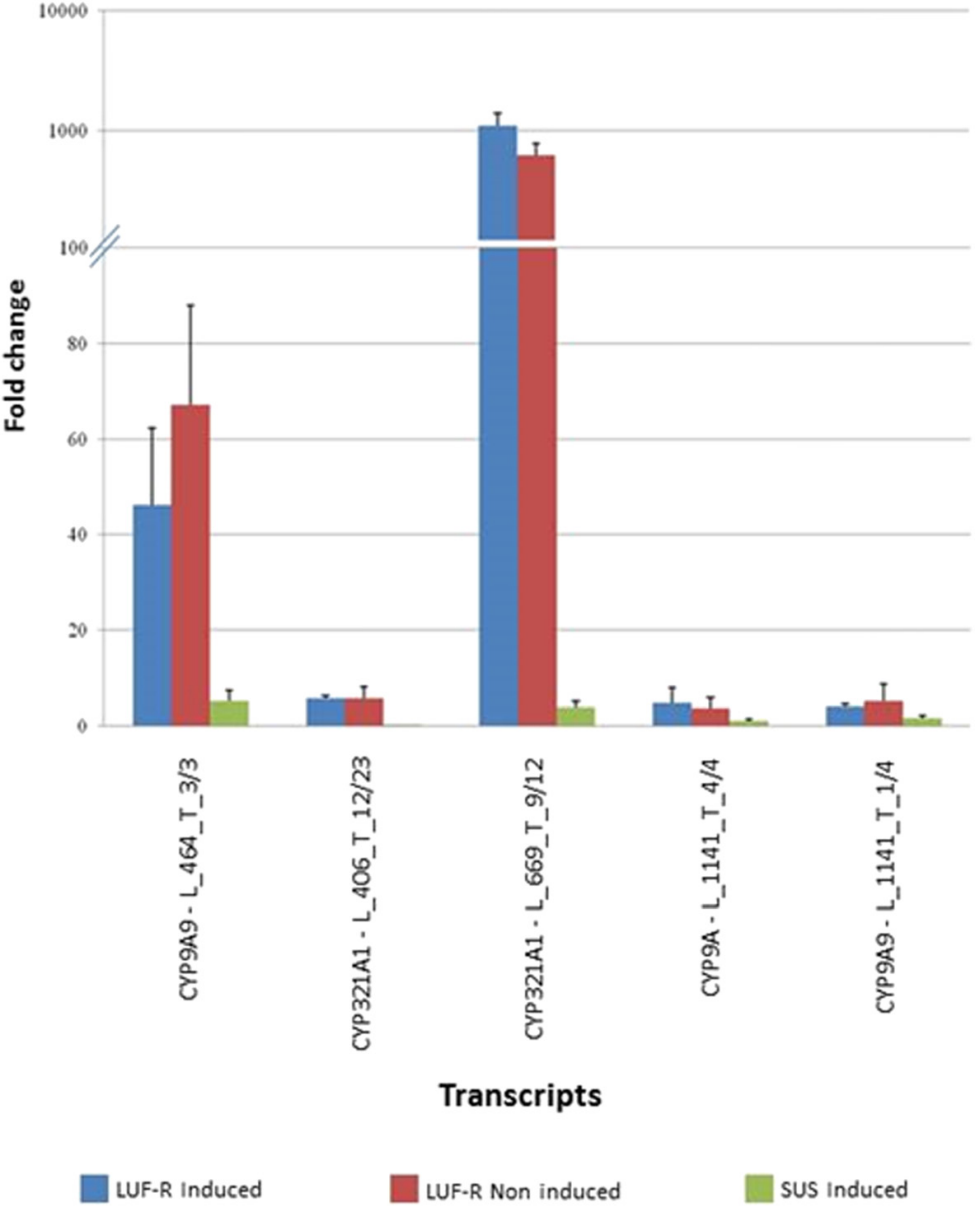
qPCR analysis of selected CYP transcripts identified as differentially expressed in a broad RNA-Seq analysis of the larval transcriptome of susceptible (LUF-S) and resistant (LUF-R) strains of *S. frugiperda* to lufenuron, exposed (induced) or not (non-induced) to lufenuron treatment. Expression of the selected genes is provided as fold change (ΔΔCt) using their expression at the LUF-S, non-induced larvae as a reference

The differential expression patterns between LUF-S and LUF-R larvae were validated by the relative expression obtained by qPCR using a selected group of transcripts (Fig. 7). All of the transcripts had higher gene expression in the LUF-R as compared to the LUF-S larvae, especially the transcripts CYP9A9 - L_464_T_3/3 and CYP321A1- L_669_T_9/12, which exhibited fold changes of 45 and 900 times, respectively.

## Discussion

Insect resistance is an evolutionary phenomenon arising from the continuous exposure of a population to the selective pressure represented by the indiscriminate use of insecticides [12]. Currently, the main mechanisms associated with resistance development are mutations in the target sites of insecticides, the intermediation of the metabolism of insecticides by detoxification enzymes and tegumental changes that limit insecticide penetration [13].

Next-generation sequencing (NGS) technologies have brought great advances for genomic studies in non-model organisms. These technologies provide a large amount of data at a low cost [14], increasing the possibility of recovering important biological information from transcriptomes [15]. Therefore, this information is of great importance, given that events such as insect resistance to insecticides are biologically complex phenomena related to adaptive processes, such as mutations and metabolic processes, that are vital for organism maintenance [12].

The *de novo* assembly of the transcriptome we performed allowed the evaluation of the differential expression between LUF-R and LUF-S larvae of *S. frugiperda*. A large number of differentially expressed transcripts were observed in between LUF-R and LUF-S larvae. To date, however, most of these transcripts have not been associated with the molecular mechanisms involved with insect resistance to insecticides. These results are similar to those reported for chlorantraniliprole-susceptible and resistant strains of *Plutella xylostella* (Lepidoptera: Plutellidae) [16]. In this case, 1,215 differentially expressed transcripts were identified, many of which were associated with metabolic pathways different from those clearly involved with the detoxification processes or of target sites of the insecticide. As an example, we observed severe down regulation of a eukaryotic translation initiation factor (L_194_T_3/3 transcript) in LUF-R as compared to LUF-S larvae, with a fold change of 1,974. Eukaryotic translation initiation factors have conserved functions related to translation events, mRNA recruitment and regulation of the cellular machinery for protein synthesis [17]. Therefore, significant changes in the expression of this transcript can be related to the strong regulation of post-transcriptional events in protein synthesis. Conversely, the L_1097_T_4/4 transcript, which is associated with the ubiquinol-cytochrome c reductase complex and linked to electron transport in the cellular respiration process [18], was the up-regulated transcript with the highest fold change (620) observed in the LUF-R strain. Regulation of genes that affect the post-transcriptional gene expression and post-translational protein modification, as the translational initiation factors and genes involved in protein ubiquitination, suggests that such mechanisms may also be involved in the observed resistance of *S. frugiperda* to lufenuron as demonstrated in the response of other organisms exposed to toxicants [19–24].

Despite a great portion of the differentially expressed transcripts was not functionally characterized, our results reveal the complexity of adaptive processes resulting from the selection pressure from the continuous exposure to lufenuron. The increase in the number of genomic-based studies associated with insect resistance has strengthened the notion that insect resistance to various insecticides can be related to polygenic and/or epigenetic factors [25], as is the case of the strain we have analyzed [26].

There have been a number of studies looking at the mode of action and on the possible resistance mechanisms of insects to benzoylphenylureas, including lufenuron, but no mechanism has been clearly demonstrated yet [27]. Many studies have argued that resistance could arise from an elevated activity of the enzymes involved in chitin processing [5, 27–29], but we did not observe significant changes in the expression of enzymes involved either in chitin synthesis (chitin synthases) or chitin degradation (chitinases). Some have also argued that resistance could be involved with the production of cuticle proteins [27]. In fact, we did detect up-regulation of transcripts associated with RR-1 cuticle proteins, L_1615_T_6/8 (cuticular protein isoform a), L_13_T_1/14 (cuticular protein rr-1 motif 46), L_13_T_11/14 (cuticular protein rr-1 motif 46) and L_623_T_2/4 (cuticle protein), but all of them are proteins associated with more flexible regions of the cuticle [30]. These results agree with those observed for diflubenzuron-treated *Tribolium castaneum* (Coleoptera: Tenebrionidae), as no changes in the expression levels of chitin synthases and chitinases were observed, although significant changes in the expression of cuticle proteins could be detected [27]. Our findings and the existing data available suggest that genes involved in chitin metabolism, modification and degradation are not the targets of benzoylphenylureas. Despite of the resistance of *Tetranychus urticae* to etoxazole was associated with mutations in conserved regions of the chitin synthase gene [31], we did not detect mutations in this gene in the LUF-R strain of *S. frugiperda*.

Alterations in the expression levels or mutations in the lufenuron receptor could also explain the resistance to lufenuron observed in the LUF-R strain of *S. frugiperda*. The lufenuron receptor remains to be described, but Abo-Elghar et al. [28] indicated the ABCC transporter sulfonylurea receptor (Sur) as the receptor for benzoylphenylurea due to the structural similarities of benzoylphenylurea and sulfonylurea. However, we did not detect any ABCC transporter or sulfonylurea receptor-like transcript in our transcriptomic analysis. Yet, the role of Sur as a sulfonylurea has been recently challenged, as this receptor was demonstrated to be dispensable for chitin synthesis in *Drosophila melanogaster* (Diptera: Drosophilidae), suggesting on the existence of an alternative sulfonylurea-sensitive ABC transporter to be involved with chitin synthesis and cuticle formation [32]. Therefore, we discarded this receptor as a possible mechanism of resistance in the LUF-R *S. frugiperda* strain.

In vitro studies have indicated benzoylphenylureas act through inhibition of the incorporation of N-acetylglucosamine (GlcNAc) into chitin in an ecdysteroid-dependent manner [33]. We did not find consistent changes in the expression of ecdysteroidogenic genes in between LUF-S and LUF-R strains of *S. frugiperda* that would suggest regulation of ecdysteroid synthesis, but we did observed a lower expression of ecdysteroid-22-kinase. This enzyme is involved in ecdysteroid inactivation in silkworm adult ovaries [34], suggesting that LUF-R larvae would retain higher titers of active ecdysteroids. Nevertheless, we did not observe any indication on differential production or incorporation of UDP-N-acetylglucosamine into chitin, as no changes in gene expression of UDP-N-acetylglucosamine diphosphorylases (GlcNAc production) or of chitin synthase (GlcNAc incorporation) were detected. Altogether, we did not find enough support to suggest the ecdysteroid-mediated regulation of GlcNAc synthesis or incorporation into chitin as a possible mechanism of resistance for the lufenuron-resistant *S. frugiperda* strain.

It has been demonstrated that benzoylphenylureas may affect cuticle formation in a concentration-dependent manner, with lower doses affecting cuticle thickness and microfibril orientation, while higher doses completely disrupt chitin synthesis [35]. The reported effects of benzoylphenylurea on the orientation and synthesis of chitin in the cuticle suggest an effect through disruption of the circadian clock involved in chitin secretion and deposition during cuticle formation [36], but we did not find evidence on the participation of clock genes as a mechanism of resistance to lufenuron in the LUF-R strain of *S. frugiperda* as well. We did though observe UDP-glucosyltransferases that were highly expressed in the LUF-R strain (L_1390_T_6/9 and L_1390_T_6/9), an enzyme that is involved with the process of chitin synthesis. However, recent progress due to large scale genomic sequencing has demonstrated UDP-glucosyltransferases form a multigenic family in insects [37, 38]. The diversity of UDP-glucosyltransferases in lepidopterans have also indicated their contribution in the process of detoxification through glycosylation [38, 39], and there are several indications in which an increase in the expression level of UGTs have been related to insect resistance to insects such DDT [40] and carbamate [41]. However, one other UDP-glucosyltransferase (L_492_T_3/8) highly similar to UGT40R3 was down regulated in the LUF-R strain, but UGT40R3 has been reported to be an UDP-glucosyltransferases exclusively associated with chemoreceptors present in insect antennae [42].

Our differential expression analysis between LUF-S and LUF-R *S. frugiperda* larvae identified a large number of transcripts associated with P450, GSTs and CCEs, of which many were overexpressed in LUF-R larvae. Detoxification of insecticides has been widely reported as one of the main mechanisms of insect adaptations to the high selective pressure exerted by insecticides [10]. Enzymes coded by genes from cytochrome P450, GSTs and CCEs are widely associated with the resistance mechanisms of insects to pesticides because of the degradation, detoxification and/or sequestration of xenobiotics [12]. The up-regulation of P450 enzymes and carboxylases, in addition to glycosyltransferases, sulfotransferases and glutathione-S-transferases, was recently demonstrated in a functional genomic analysis of a population of *Tribolium castaneum* contaminated with diflubenzuron, corroborating their association with the detoxification of xenobiotics [27].

A higher number of P450 monooxygenases was observed among the ESTs with higher relative expression. P450 monooxygenases have been one of the main classes of enzymes associated with lepidopteran resistance to insecticides such as pyrethroids [43], organophosphates [44] and diamides [16]. Several studies have been conducted aiming to evaluate the activity of these enzymes at the toxicological level [45]. Their role in insecticide degradation has been demonstrated by observed increased mortality of resistant populations of *S. frugiperda* to pyrethroids, organophosphates and carbamates when exposed mainly to monooxygenase inhibitors [46]. The role of CYPs in lufenuron detoxification has also been suggested, as CYP12A4 was up-regulated in a population of *Drosophila melanogaster* resistant to lufenuron, suggesting that the enzymes from cytochrome P450 play a key role in the resistance against this insecticide.

The finding of multiple overexpressed CYP families in LUF-R larvae also points for their role in the resistance of *S. frugiperda* to lufenuron. CYP3, CYP4, CYP6 and CYP9 are P450 families that have been argued as one of the mechanisms involved in lepidopteran resistance to insecticides [47–49]. The high expression levels associated with the genes encoding detoxification enzymes, even in the absence of insecticide, show that the high expression of these genes occurs constitutively.

The metabolism of insecticides in insects certainly involves a series of complex metabolic processes. Even though the biochemical processes related to the detoxification are well described, there are important gaps related to the physiological and molecular mechanisms that govern the detoxification process [50]. We were able to demonstrate the overexpression of genes involved in the functionalization (phase I – *eg* CYPs) and conjugation (phase II – *eg* UDP-glucosyltransferases, GSTs), but not of genes involved in the excretion (phase III – *eg* ABC transporters) of xenobiotics in the process of lufenuron detoxification in the LUF-R strain of *S. frugiperda* [50]. This may indicate that the process of detoxification of lufenuron can mainly occur within the lumen of the gut.

The diverse gene expression profile between LUF-S and LUF-R strains indicate resistance also affects several other pathways, which may be related with the resistance fitness costs or may represent an additional mechanism to contribute with resistance of the LUF-R *S. frugiperda* to lufenuron. Therefore, there is the need for studies that provide a better understanding of the metabolic pathways adjacent to the classical detoxification pathways [12]. For many years, studies of the resistance mechanisms of insects to insecticides were scarce. However, the advance of sequencing technologies and the ability to generate large volumes of information have provided a better understanding of the mechanisms involved in the processes that lead to the resistance of insects to insecticides [16].

## Conclusions

Gene expression of LUF-R larvae of *S. frugiperda* is much higher than of LUF-S in general, and particularly of those genes involved the detoxification process, including CYP, CCE and GST. We concluded that the high abundance of highly expressed CYP genes in the resistant as compared to the susceptible strain is one of the major mechanisms involved in resistance of *S. frugiperda* to lufenuron.

## Methods

### Insect preparation

Two laboratory-selected strains of *S. frugiperda* were used for sequencing, a lufenuron-susceptible strain (LUF-S) and a lufenuron-resistant strain (LUF-R). LC_50s_ for LUF-S and LUF-R were estimated at 0.23 and 216.6 μg.mL^−1^ of lufenuron, respectively [26]. Larvae of fourth instars were fasted for 24 h. After this period, susceptible and resistant larvae were split into subgroups that were submitted or not to induction with lufenuron. Larvae were transferred to containers with an artificial diet treated superficially with 3.2 μg.mL^−1^ of lufenuron (Match 50 EC, 500 g.L^−1^ of lufenuron, emulsifiable concentrate, Syngenta Proteção de Cultivos Ltda.) diluted in distilled water added with 0.1 % Triton® and remained in contact with the food source for one hour (induced). In the case of control treatments, the artificial diet was surface-treated only with the 0.1 % Triton in water (non-induced). Therefore, larvae selected for RNA extraction and cDNA sequencing comprised four groups: induced and control LUF-S, and induced and control LUF-R.

### Tissue sampling and total RNA extraction

Total RNA was extracted from five fourth instars per treatment using Trizol® Reagent (Invitrogen™), according to the manufacturer’s instructions. Samples were macerated in liquid nitrogen and homogenized in 1 mL Trizol reagent by vortexing, followed by the addition of 200 μL. Sample was mixed by vortexing and centrifuged for 15 min at 12,000 *g*. The aqueous phase was recovered, added to 500 μL of isopropanol and centrifuged (10 min x 12,000 *g*). The pellet obtained was washed in 1 mL of 75 % ice-cold ethanol, and the sample was centrifuged once again (5 min x 7,500 *g*). The pellet was air-dried at room temperature and resuspended in 60 μL of sterile milliQ water. Residual DNA was eliminated after treatment with 9 U of RNAse-free DNAse. The supernatant was recovered and added to 300 μL of Trizol and 200 μL of chloroform. Sample was agitated and centrifuged for 10 min at 13,000 *g*. The supernatant was discarded, the pellet was recovered in 300 μL of chloroform, and the solution was centrifuged for 5 min at 13,000 *g*. The supernatant was recovered, 1 mL of 100 % ethanol was added and the sample centrifuged again (10 min x 13,000 *g*). The pellet obtained was air-dried at room temperature and resuspended in DEPC-treated water. RNA quantity and quality were assessed in a Picodrop-Spectrophotometer 4.0.4.0.

The mRNA population in each sample was enriched by removing ribosomal RNA (rRNA) using the commercial system RiboMinus® Eukaryote Kit (Invitrogen™), following the manufacturer’s instructions. mRNA-enriched samples of LUF-S and LUF-R larvae of *S. frugiperda* were used for cDNA library preparation using the TruSeq Stranded mRNA Library Prep Kit, and sequencing in a HiSeq 1000® (Illumina^©^) platform at the Laboratory of Animal Biotechnology, Department of Animal Science, ESALQ, University of São Paulo, a sequencing service provider.

### *De novo* assembly

The *paired-end* protocol was used and originated reads of approximately 100 bp for the sequencing of cDNA libraries. The reads obtained from the Illumina HiScan 1000® platform sequencing were filtered for removing bases with quality scores lower than 30 (where a Phred score of 30 corresponds to a 0.1 % expected error rate) at both the 5′and 3′ends and used for the assembly of a single reference transcriptome.

Then, aiming to maximize the computational performance, the duplicate reads were excluded, minimizing the time used for the *de novo* assembly of the transcriptome. The *paired-end* obtained were subjected to exploratory analysis using the *Velvetoptimiser* version 2.2.5 (https://github.com/tseemann/VelvetOptimiser) [51] to evaluate the diversity of transcripts assembled in different motif lengths (*k-*mers) [52]. Therefore, assemblies were obtained for all odd sizes of *k-*mers, varying from 19 to 61, following [51]. Assemblies with *k*-mers of 23, 25, 47, 53 and 55 obtained with the software Velvet version 1.2.10 (https://github.com/dzerbino/velvet/tree/master) were used to obtain a reference transcriptome. The concatenation of *contigs* of the assemblies obtained from the different *k-*mers used was performed in *Oases* version 0.2.08 (www.ebi.ac.uk/~zerbino/oases/) [53]. Transcripts sharing 95 % similarity were grouped using the *CD-hit* version 4.6 (https://github.com/weizhongli/cdhit) [54] to reduce the redundancy of the final assembly.

### Functional annotation

The obtained transcripts were annotated after a similarity search against the NCBI non-redundant database using the BLASTx algorithm [55] available with the BLAST2GO software [56], with a cutoff value of 10^−3^. Enzyme classification (EC) codes and the annotation of metabolic pathways (KEGG - Kyoto Gene and Genomes) were generated from the direct mapping of the GO terms with their equivalent enzyme codes [57]. For functional classification, the consensus sequences were compared to those from the Interpro protein signature databases using the InterproScan with a cutoff of 10^−5^ [58].

### Differential expression between the LUF-S and LUF-R *S. frugiperda* strains

The differential expression between the LUF-R and LUF-S strains of *S. frugiperda* was evaluated by determining the number of reads per kilobase of transcript per million reads (RPKM) mapped against the *de novo* assembled reference transcriptome earlier described, using only reads that counted against one target reference transcript following the remaining default parameters in the CLC Genomics Workbench software (QIAGEN Company). The experimental design comprised of two groups, the LUF-R or the LUF-S strain, each one counting with the subgroups induced and non-induced. To obtain a high specificity for the results, only the reads similar to a single transcript were computed, with a minimum similarity of 80 % and a read length alignment higher than 90 %. The mean RPKM values obtained for the resistant strain (induced and non-induced) were compared to those of the susceptible strain (induced and non-induced) by the *t*-test (*p* < 0.05), using the tools available in the CLC Genomics Workbench software (QIAGEN Company). Only transcripts with significant differences in the expression levels based on the discriminative significance values (*p* ≤ 0.05) and relative expression (fold change >4) were considered differentially expressed.

The validation of the comparative analysis using RNA-seq data between LUF-R and LUF-S *S. frugiperda* strains was performed for a set of differentially expressed transcripts by using quantitative real-time PCR (qPCR). Transcripts possibly associated with the mechanisms of resistance to lufenuron, such as those belonging to cuticle metabolism and metabolic detoxification enzymes were randomly selected as candidate genes (Table 1).

**Table 1:** Primer sets used in qPCR reactions for validation of the RNA-Seq analysis

Transcript		Sequence (5′- 3′)
L_464_T_3/3	Forward	CAATGCAATTCCTTGGACCAA
	Reverse	GCACACCATCGATCCAATGA
L_406_T_12/23	Forward	TCGGTGAGAGGTATGCCAAAT
	Reverse	AAGTTTCGCAAGACGTGCACTA
L_669_T_9/12	Forward	CAAACCAGCCTGCACCTGTA
	Reverse	GGGCAACAGGACGTGTATAGG
L_1141_T_4/4	Forward	CCATCGAGGTTGTGCAAAAA
	Reverse	CCTCAGCCAGTGTCCTCCAT
L_1141_T_1/4	Forward	CAATCCGCTCGCGTACATG
	Reverse	AATCTTGACCCAATGCAATTCC
GAPDH	Forward	CGGTGTCTTCACAACCACAG
	Reverse	TTGACACCAACGACGAACAT

### qPCR validation of candidate genes

For validation of our differential expression analysis between LUF-S and LUF-R strains of *S. frugiperda*, 2 μg of RNA treated with RNAse-free DNAse obtained from each treatment was used in a reverse transcription reaction to produce cDNA. For the reverse transcription reaction, the commercial system ImProm-II™ Reverse Transcription System (Promega©) was used according to the manufacturer’s instructions. The cDNA was stored at −20 °C until use.

qPCR reactions consisted of 0.4 μg of cDNA, 12.5 μL of Maxima SYBR Green/ROX qPCR Master Mix® (Thermo Scientific©), 0.6 μM of each of the primers (Table 1) and 10.3 μL of water, totaling a final volume of 25 μL. The primers for each selected target were designed using the Primer Express® software (Life Technologies©), based on the sequences of the *S. frugiperda* transcriptome. The amplification reactions were performed in a ViiA™ 7 Real Time PCR System (Applied Biosystems®, Life Technologies^©^) platform. The thermocycling conditions used were as follows: 2 min of preheating at 50 °C, 10 min for initial denaturation at 95 °C, followed by 35 cycles at 95 °C for 15 s, 60 °C for 30 s and 72 °C for 30 s. The dissociation curves were analyzed to assess the specificity of the amplification. The standardization of the amplification was performed using glyceraldehyde 3-phosphate dehydrogenase (GAPDH) [59].

The experiment followed a completely randomized design, composed of four treatments similar to those used for the sequencing, containing three biological replicates (each replicate consisted of five larvae); each replicate was analyzed in technical triplicates. Differences in gene expression between samples analyzed were determined following the ΔΔCt method [60].

### Availability of supporting data

All Illumina data have been deposited in NCBI’s Sequence Read Archive (SRA) under accession number PRJNA299878.

## Additional files

Additional file 1:
**Pre-processing of the data resulting from the sequencing of susceptible and lufenuron-resistant strains of** ***S. frugiperda.*** (DOCX 14 kb) Additional file 2:
**Exploratory analysis of the** ***de novo*** **assembly of the larval transcriptome of lufenuron-susceptible (LUF-S) and lufenuron-resistant (LUF-R) strains of** ***Spodoptera frugiperda.*** (DOCX 14 kb) Additional file 3:
**Summary of the** ***de novo*** **assembly of the transcriptome of susceptible and lufenuron-resistant strains of** ***S. frugiperda.*** (DOCX 14 kb) Additional file 4:
**Size distribution of the transcripts of the reference transcriptome of susceptible and lufenuron-resistant larvae of** ***S. frugiperda.*** (DOCX 19 kb) Additional file 5:
**Distribution of the homology values of the transcripts of the** ***de novo*** **transcriptome of** ***S. frugiperda*** **obtained after database search (nr)(NCBI).** (DOCX 15 kb) Additional file 6:
**Distribution of alignments of the transcripts of the** ***de novo*** **transcriptome of** ***S. frugiperda*** **by species obtained via BLAST.** (DOCX 16 kb) Additional file 7:
**Distribution of gene ontologies (GO) assigned to the transcriptome of susceptible and lufenuron-resistant strains of** ***S. frugiperda.*** (DOCX 225 kb) Additional file 8:
**Differentially expressed transcripts between resistant and susceptible strains of** ***S. frugiperda*** **to lufenuron.** (XLS 95 kb)

## Abbreviations

Bt: *Bacillus thuringiensis*
CCE: carboxylase
CYP: cytochrome P450
DDT: 1,1,1-trichloro-2,2-di(4-chlorophenyl)ethane
GAPDH: Glyceraldehyde 3-phosphate dehydrogenase
GO: Gene Ontology
GST: Glutathione S-transferase
IPM: Integrated Pest Management
KEGG: Kyoto Gene and Genomes
LC50: lethal concentration 50
LUF-R: Strain Resistance to lufenuron
LUF-S: Strain Susceptible to lufenuron
NCBI: National Center for Biotechnology Information
NGS: Next Generation Sequencing
qPCR: Quantitative real-time PCR
RPKM: Reads per kilo base per million mapped reads
SUR: Sulfonylurea receptor
UGT: UDP-glycosyltransferase
