# Supplementary material for: Comparative transcriptome analysis of lufenuron-resistant and susceptible strains of Spodoptera frugiperda (Lepidoptera: Noctuidae)
Source: BMC Genomics. 2015 Nov 21;16:985. doi: 10.1186/s12864-015-2183-z (PMC4654862; doi:10.1186/s12864-015-2183-z)
Supplement: Additional file 1: — Pre-processing of the data resulting from the sequencing of susceptible and lufenuron-resistant strains of S. frugiperda. (DOCX 14 kb) [file 12864_2015_2183_MOESM1_ESM.docx]

Additional file 1 – Pre-processing of the data resulting from the sequencing of susceptible and lufenuron-resistant strains of *S. frugiperda*

| **Raw data** |  |
| --- | --- |
| Number of reads (*single-end*) | 68,027,513 |
| Total number of nucleotides | 6,802,751,300 |
|  |  |
| **After filtering** |  |
| Number of reads (*single-end*) | 52,280,210 |
| Total number of nucleotides | 4,705,218,900 |
|  |  |
| **After removal of duplicate reads** |  |
| Number of reads (*single-end*) | 19,969,569 |
| Total number of nucleotides | 1,797,261,210 |

* Illumina 1.9 sequencing
