# Supplementary material for: Comparative transcriptome analysis of lufenuron-resistant and susceptible strains of Spodoptera frugiperda (Lepidoptera: Noctuidae)
Source: BMC Genomics. 2015 Nov 21;16:985. doi: 10.1186/s12864-015-2183-z (PMC4654862; doi:10.1186/s12864-015-2183-z)
Supplement: Additional file 2: — Exploratory analysis of the de novo assembly of the larval transcriptome of lufenuron-susceptible (LUF-S) and lufenuron-resistant (LUF-R) strains of Spodoptera frugiperda. (DOCX 14 kb) [file 12864_2015_2183_MOESM2_ESM.docx]

Additional file 2 – Exploratory analysis of the de novo assembly of the larval transcriptome of lufenuron-susceptible (LUF-S) and lufenuron-resistant (LUF-R) strains of *Spodoptera frugiperda*

| ***k*-mer** | **Transcripts (n)** | **N50** | **Size of larger transcripts** | **Total number of assembled bases** | **nº of transcripts**  **> 1k** | **Total number of bases in transcripts> 1k** |
| --- | --- | --- | --- | --- | --- | --- |
| 19 | 15,563 | 299 | 1,666 | 4,774,489 | 46 | 53,084 |
| 21 | 20,084 | 325 | 1,827 | 6,602,807 | 160 | 190,429 |
| 23 | 21,295 | 330 | 1,930 | 7,081,839 | 196 | 237,443 |
| 25 | 21,972 | 329 | 1,930 | 7,315,647 | 203 | 246,328 |
| 27 | 22,913 | 327 | 2,132 | 7,589,209 | 210 | 253,358 |
| 29 | 23,830 | 325 | 2,759 | 7,874,519 | 215 | 264,032 |
| 31 | 24,726 | 324 | 2,759 | 8,153,945 | 238 | 292,700 |
| 33 | 25,751 | 322 | 2,759 | 8,464,652 | 267 | 327,038 |
| 35 | 26,529 | 323 | 2,759 | 8,728,509 | 264 | 323,776 |
| 37 | 27,700 | 321 | 3,076 | 9,090,666 | 268 | 331,771 |
| 39 | 28,761 | 322 | 2,452 | 9,452,896 | 313 | 382,965 |
| 41 | 29,732 | 324 | 2,452 | 9,808,008 | 353 | 429,227 |
| 43 | 31,031 | 324 | 2,469 | 10,254,450 | 370 | 456,786 |
| 45 | 32,200 | 325 | 2,469 | 10,664,873 | 397 | 491,366 |
| 47 | 33,023 | 327 | 2,423 | 10,979,048 | 429 | 530,833 |
| 49 | 33,819 | 326 | 2,520 | 11,243,998 | 437 | 536,766 |
| 51 | 34,688 | 325 | 2,423 | 11,485,845 | 415 | 513,622 |
| 53 | 34,841 | 327 | 2,427 | 11,566,833 | 428 | 526,515 |
| 55 | 35,000 | 326 | 2,594 | 11,619,179 | 434 | 533,791 |
| 57 | 35,369 | 324 | 2,594 | 11,693,969 | 416 | 510,464 |
| 59 | 34,736 | 326 | 2,800 | 11,497,603 | 387 | 480,907 |
| 61 | 34,341 | 326 | 2,594 | 11,351,874 | 371 | 457,984 |
