# Supplementary material for: Comparative transcriptome analysis of lufenuron-resistant and susceptible strains of Spodoptera frugiperda (Lepidoptera: Noctuidae)
Source: BMC Genomics. 2015 Nov 21;16:985. doi: 10.1186/s12864-015-2183-z (PMC4654862; doi:10.1186/s12864-015-2183-z)
Supplement: Additional file 3: — Summary of the de novo assembly of the transcriptome of susceptible and lufenuron-resistant strains of S. frugiperda. (DOCX 14 kb) [file 12864_2015_2183_MOESM3_ESM.docx]

Additional file 3 – Summary of the *de novo* assembly of the transcriptome of susceptible and lufenuron-resistant strains of *S. frugiperda*

|  | **Assembly** |
| --- | --- |
|  |  |
| **Number of transcripts** | 18,506 |
| **Min. Size** | 100 |
| **Max. Size** | 6,517 |
| **Total bases** | 14,337,437 |
| **Size > 1000** | 4,945 |
| **Mean size of transcripts** | 774.75 |
| **N50** | 996 |
| **N90** | 411 |
