# Supplementary material for: Comparative transcriptome analysis of lufenuron-resistant and susceptible strains of Spodoptera frugiperda (Lepidoptera: Noctuidae)
Source: BMC Genomics. 2015 Nov 21;16:985. doi: 10.1186/s12864-015-2183-z (PMC4654862; doi:10.1186/s12864-015-2183-z)
Supplement: Additional file 6: — Distribution of alignments of the transcripts of the de novo transcriptome of S. frugiperda by species obtained via BLAST. (DOCX 16 kb) [file 12864_2015_2183_MOESM6_ESM.docx]

Additional file 6 – Distribution of the alignments of the transcripts of the *de novo* transcriptome of *S. frugiperda* by species obtained via BLAST
