# Supplementary material for: Comparative transcriptome analysis of lufenuron-resistant and susceptible strains of Spodoptera frugiperda (Lepidoptera: Noctuidae)
Source: BMC Genomics. 2015 Nov 21;16:985. doi: 10.1186/s12864-015-2183-z (PMC4654862; doi:10.1186/s12864-015-2183-z)
Supplement: Additional file 7: — Distribution of gene ontologies (GO) assigned to the transcriptome of susceptible and lufenuron-resistant strains of S. frugiperda. (DOCX 225 kb) [file 12864_2015_2183_MOESM7_ESM.docx]

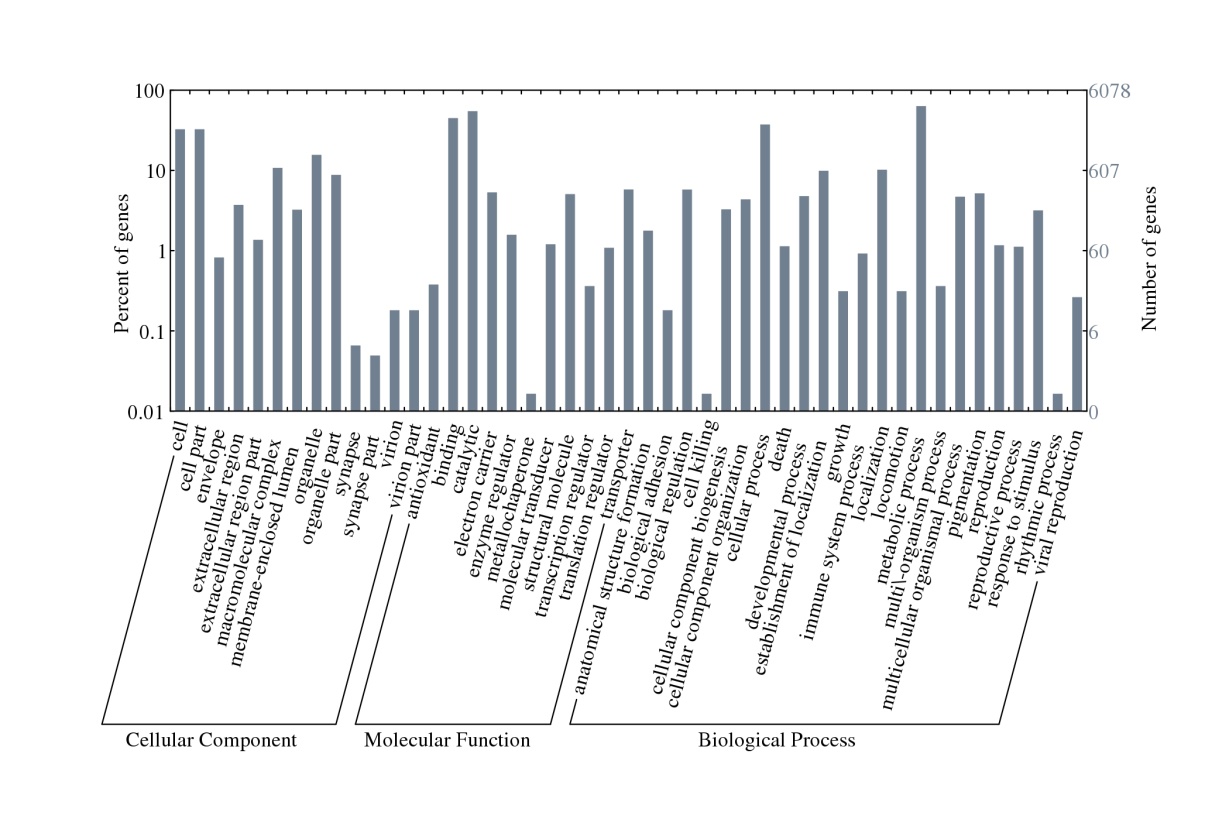


Additional file 7 – Distribution of gene ontologies (GO) assigned to the transcriptome of susceptible and lufenuron-resistant strains of *S. frugiperda*
